# A plant-expressed conjugate vaccine breaks CD4^+^ tolerance and induces potent immunity against metastatic Her2^+^ breast cancer

**DOI:** 10.1080/2162402X.2016.1166323

**Published:** 2016-04-22

**Authors:** Warayut Chotprakaikiat, Alex Allen, Duc Bui-Minh, Elena Harden, Jantipa Jobsri, Federica Cavallo, Yuri Gleba, Freda K. Stevenson, Christian Ottensmeier, Victor Klimyuk, Natalia Savelyeva

**Affiliations:** a Cancer Sciences Unit, Faculty of Medicine, University of Southampton, Southampton, UK; b Icon Genetics, Halle, Germany; c Oral Biology Department, Naresuan University, Phitsanulok, Thailand; d Department of Molecular Biotechnology and Health Sciences, Molecular Biotechnology Center, University of Turin, Turin, Italy

**Keywords:** Antibody, breast cancer, CD4^+^ T helper cells, plant-based bio-manufacturing, vaccines

## Abstract

Passive antibody therapy for cancer is an effective but costly treatment modality. Induction of therapeutically potent anticancer antibodies by active vaccination is an attractive alternative but has proven challenging in cancer due to tolerogenic pressure in patients. Here, we used the clinically relevant cancer target Her2, known to be susceptible to targeting by antibody therapy, to demonstrate how potent antibody can be induced by vaccination. A novel 44kD Her2 protein fragment was generated and found to be highly effective at inducing anti-Her2 antibody including trastuzumab-like reactivities. In the tolerant and spontaneous BALB-neuT mouse model of metastatic breast cancer this Her2-targeting vaccine was only effective if the fragment was conjugated to a foreign immunogenic carrier; Fragment C of tetanus toxin. Only the conjugate vaccine induced high affinity anti-Her2 antibody of multiple isotypes and suppressed tumor development. The magnitude of CD4^+^ T-cell help and breadth of cytokines secreted by the CD4^+^ T helper (Th) cells induced to the foreign antigen was critical. We used a highly efficient plant-based bio-manufacturing process for protein antigens, magnICON, for vaccine expression, to underpin feasibility of future clinical testing. Hence, our novel Her2-targeting conjugate vaccine combines preclinical efficacy with clinical deliverability, thus setting the scene for therapeutic testing.

## Introduction

In the last two decades immunotherapy has evolved into a breakthrough therapeutic modality for combating cancer.^1^ Passive monoclonal antibodies (Mabs) targeting antigens on cancer cells or stimulating antitumor immunity through the release of blocking signals on T cells have shown significant clinical benefits, extending survival of patients with primary and metastatic cancers.^2,3^ Using vaccines to educate the patient's immune system to recognize tumor antigens is also emerging as a clinically relevant concept. The first therapeutic vaccine, Provenge^4^ was approved by the FDA in 2010 and several cancer vaccines are in late stage clinical evaluation.^5-9^

**Figure 1.**
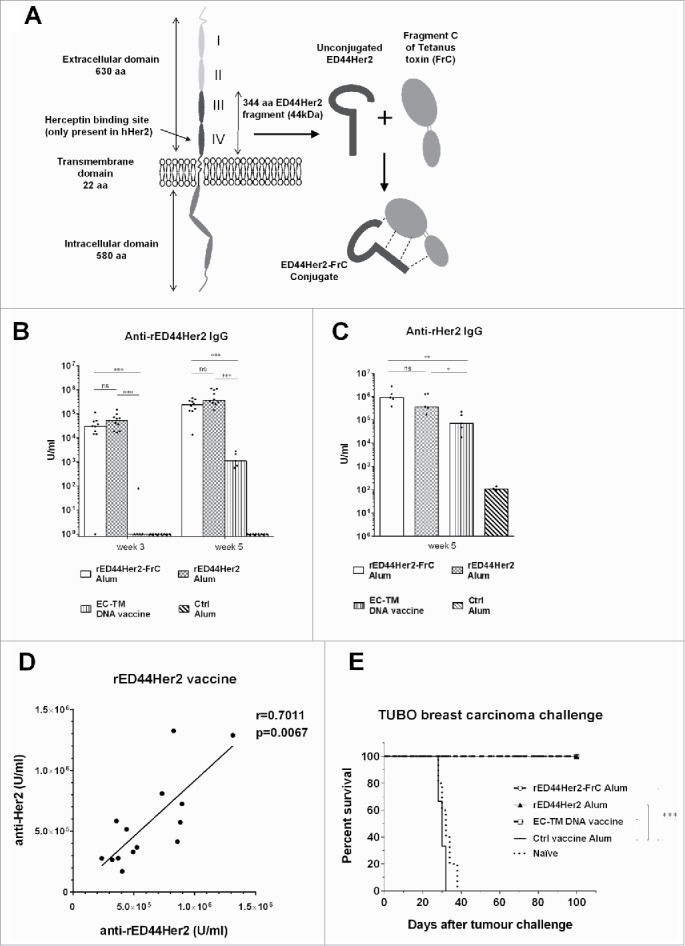
rED44Her2 and rED44Her2-FrC conjugate vaccines induce potent humoral immunity against rED44Her2 and native rHer2, and protect against challenge with the TUBO mammary carcinoma. (A) Schematic representation of the Her2 molecule (highlighting the ED44Her2 fragment) and the ED44Her2-FrC conjugate vaccine design. Diagram is representative of both the rat and human forms of Her2 and ED44Her2. (B) BALB/c wild type mice were primed and boosted 3 weeks later with rED44Her2, rED44Her2-FrC or the plant expressed control vaccine, all in alum, or the EC-TM DNA vaccine (n = 5 per group). Antibody specific for the rED44Her2 fragment was measured by ELISA. Serum was collected after priming, at week 3, and at week 5 which was 2 weeks after the boost. (C) Antibody able to bind native rHer2 expressed on the surface of TUBO cells was measured by flow cytometry at week 5 and quantified using internal standards. (D) Correlation between the levels of anti-rED44Her2 antibody, as measured by ELISA, and anti-rHer2 antibody, as measured by binding to native rHer2 at the cell surface. Data from the rED44Her2 vaccine at week 5. r = the Spearman's rank correlation coefficient. (E) 6 weeks after the booster injection (week 9) mice were challenged with the transplantable TUBO mammary carcinoma, and culled when tumors reached 15 mm diameter. In (B) and (C) each bar represents medians with individual mice displayed as dots. Mann–Whitney statistics are shown. Log-rank (Mantel–Cox) test results shown in (E). ns = *p* > 0.05, *p < 0.05, **p < 0.01, ***p < 0.001. For (B) and (D) data from two experiments has been combined. For (C) and (E) one representative experiment of three is shown.

Human epidermal growth factor receptor Her2, also known as Neu, ErbB-2, or p185, has emerged as an important target for cancer immunotherapy. Activation of Her2 through gene amplification or mutations occurs in many solid tumors including 20–30% of breast and ovarian cancers, and is linked to more aggressive disease and poor prognosis.^10-12^ Like other members of the epidermal growth factor receptor family, Her2 is a receptor tyrosine kinase composed of an extracellular (EC) domain, a transmembrane (TM) domain and an intracellular (IC) domain which interacts with downstream signaling molecules (Fig. 1A). Through homodimerisation, or heterodimerisation with other family members, Her2 can transduce deregulated signals responsible for neoplastic behavior of cells.^13^

A Mab targeting Her2, trastuzumab (Herceptin) alone or linked with a cytotoxic drug DM1 (trastuzumab emtansine) has had significant impact on survival rates from Her2 positive metastatic breast cancer.^14,15^ More recently a second anti-Her2 Mab, pertuzumab, was approved in combination with Herceptin and chemotherapy for untreated Her2/neu positive metastatic breast cancer patients; the combination achieved significant improvement in progression free and overall survival.^16^

Clearly Her2 is an excellent target for antibody attack but for passive Mab therapy a number of limitations arise, including tumor recurrence within an year of treatment, reflecting development of antibody resistance.^14^ Additionally, the need for repeated dosing to maintain protective antibody levels imposes a significant burden on healthcare. Active immunization, i.e. vaccination, can induce durable polyclonal antibody responses, thus eliminating the need for multiple re-infusions and reducing development of antibody resistance. Hence, vaccination approaches for targeting Her2 (whole-cell vaccines, peptides, DNA encoding Her2 regions, viral vaccines and Her2 protein fragments) have been developed and tested in human clinical trials.^17^ In a recent phase I clinical trial, a protein vaccine consisting of the Her2 EC domain and a portion of the IC domain was given in combination with a complex adjuvant that included TLR agonists. Anti-Her2 antibodies were detectable, but showed limited anti-Her2 inhibitory effects *in vitro*.^18,19^

A critical factor for induction of potent antibody responses is the availability of cognate CD4^+^ T-cell help, which drives B cells through a germinal center reaction where selection based on high affinity for antigen occurs, along with antibody isotype-switch.^20^ This ensures the subsequent antibody response is of high quality, through improved antigen binding and enhanced effector function via changing the antibody Fc portion. Once the antibody response is induced, T-cell help remains critical as high-affinity memory responses cannot be maintained in its absence.^21,22^ In cancer, this T-cell help may not be inducible by cancer antigens themselves as multiple immune tolerogenic mechanisms exist to dampen induction of CD4^+^ Th cells to these self-proteins.^23^

To overcome tolerogenic mechanisms, we have developed DNA vaccines incorporating the target antigen linked to a non-toxic portion of Tetanus Toxin FrC^24^ or its subdomain Dom.^25^ This approach allowed the capture of CD4^+^ T-cells from the non-tolerized repertoire against foreign antigens, to enable induction of immunity against cancer targets.^25^ These fusion DNA vaccine designs have demonstrated induction of antibody and CD8^+^ T-cell responses in several phase I–II clinical trials for both hematological^26^ and solid tumor targets^27^ with suggestion of clinical benefit.^62^ DNA vaccines avoid the step of protein expression *in vitro*, and can induce high levels of cytotoxic T cells,^25,27,^^28^ but the small amounts of protein produced *in vivo* may not be ideal for activating B cells to produce optimal levels of antibody.

We have now harnessed this principle of linked T-cell help and exploited it for a protein vaccine to overcome tolerance to Her2. We generated a novel 44kD protein fragment of the Her2 EC domain (ED44Her2) to direct Her2 antibody specificity and this was conjugated to FrC (ED44Her2-FrC). Both vaccine subunits were expressed using plant-based bio-manufacturing for proteins using magnICON methodology.^29,30^ This process has emerged in recent years as an efficient process for achieving high levels of protein expression using plant viral vectors to drive gene expression in non-transgenic plants.^29,31^ Since targeting Her2 with antibodies is therapeutically relevant, for vaccine delivery we used formulation with alum which aimed to focus the immune response on antibody induction and is known not to induce functional CD8^+^ cytotoxic T cells.^32,33^ We demonstrate that this ED44Her2-FrC vaccine design is able to induce potent antibody and overcome tolerance in a preclinical model of metastatic breast cancer. The magnitude and breadth of cytokines produced by CD4^+^ Th cells engaged by conjugation to FrC resulted in generation of high-affinity protective anti-Her2 antibody. Hence, the novel vaccine has potential to become a plausible therapeutic option for Her2 positive cancers.

## Results

### Her2 vaccine design and expression

To target Her2, we chose the 344 aa fragment that spans domains III and IV of the Her2 EC domain, without incorporation of the TM domain (Fig. 1A). This is the region targeted previously by both trastuzumab^34^ and vaccination.^35^ We expressed this novel 44kD ED44Her2 protein in *Nicotiana benthamiana* plants using the magnICON system.^29^ The relatively high content of cysteine residues in the ED44Her2 fragment (29 cysteine residues or 8.5% of total amino acids) caused a certain “stickiness” of the fragment, but this did not have any significant impact on downstream processing. Both the rat and human forms of ED44Her2 were generated. This allowed testing in both the spontaneous breast cancer model BALB-neuT that is driven by the activated rat Her2 transgene,^36^ or the transplantable D2F2/E2 mammary carcinoma which is transfected with human Her2.^37^ Plant-expressed FrC was also produced, and conjugated to either rED44Her2 (rat) or hED44Her2 (human), in order to capture non-tolerized CD4^+^ Th cells to help B cells generate potent antibody against Her2.

### Vaccines containing the rED44Her2 fragment induce antibody against native rHer2

To evaluate the ability of rED44Her2 and the rED44Her2-FrC conjugate to induce antibody responses against native Her2, we vaccinated BALB/c wild type (wt) mice with rED44Her2, rED44Her2-FrC or a plant-expressed control vaccine, in combination with the adjuvant alum. First, antibody specific for rED44Her2 was measured by ELISA and Fig. 1B demonstrates that similar levels of antibody were induced by both rED44Her2 containing vaccines after both priming and booster injections (measured at week 3 and week 5). The results were compared to the “gold standard” vaccine for this model, a DNA vaccine that encodes the entire Her2 EC plus TM domains which has been shown to induce antibody against rHer2.^38^ Here, we found the DNA vaccine induced low levels of antibody to rED44Her2 in comparison with the protein vaccines containing rED44Her2 (Fig. 1B; *p* = 0.0007 for both vaccines at both time points).

Next, we determined antibody reactivity to native rHer2 expressed on the surface of mammary carcinoma TUBO cells by flow cytometry. The antibody induced by both rED44Her2 and the rED44Her2-FrC conjugate was able to recognize native rHer2, and antibody levels between the two vaccines were similar when compared after boosting at week 5 (Fig. 1C), paralleling the findings in ELISA. Both protein vaccines induced over 5-fold higher anti-rHER2 antibody than the rEC-TM DNA vaccine (Fig. 1C; *p* = 0.0159 for rED44Her2 and *p* = 0.0079 for rED4Her2-FrC). Hence, rED44Her2 and rED44Her2-FrC were able to induce high levels of antibody against native surface expressed rHer2, indicating that the rED44Her2 protein fragment used in both vaccines folded well, mimicking the antigenic determinant of native Her2. Conjugation to FrC did not appear to affect the antigenic properties of rED44Her2.

Correlation between antibody levels measured by ELISA or against native rHer2 further supported the preservation of critical antigenic properties in the rED44Her2 fragment, with similar correlations found with both unconjugated and conjugated vaccines (Fig. 1D and Fig. S1). Furthermore, the data validated the usage of rED44Her2 for measuring responses against native rHer2 by ELISA.

We next tested the ability of the rED44Her2-based vaccines to protect against TUBO carcinoma challenge. Mice primed then boosted at week 3 were challenged at week 9 and tumor development followed. Both rED44Her2 containing vaccines were able to protect 100% of mice, as was the rEC-TM DNA vaccine, until the experiment was terminated at day 80. The control vaccine group and the naive control group developed terminal tumors between day 28 and day 38 (Fig. 1E).

### The rED44Her2-FrC conjugate vaccine overcomes tolerance and rapidly induces high levels of antibody in BALB-neuT tolerant mice

**Figure 2.**
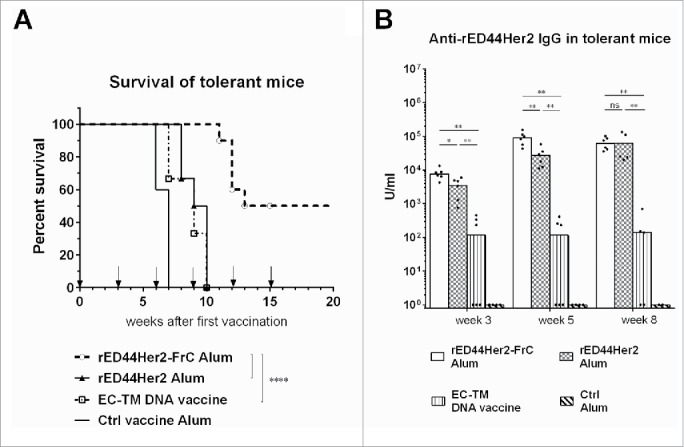
The rED44Her2-FrC conjugate vaccine affords superior protection against spontaneous mammary carcinoma in the BALB-neuT model, and induces high levels of anti-rED44Her2 antibody. (A) BALB-neuT mice aged 10 weeks were vaccinated with rED44Her2, rED44Her2-FrC or the irrelevant control protein vaccine, in alum, or the EC-TM DNA vaccine. They were then boosted every 3 weeks (see arrows on graph). Development of spontaneous tumors was observed and mice terminated when the sum total mean diameter of tumors exceeded 15 mm. (B) Serum samples from mice in (A) were tested for rED44Her2-specific antibody at the indicated time points by ELISA. In (A) log-rank (Mantel–Cox) test results are shown, and in (B) median values are plotted as bars, with Mann–Whitney statistics. Ns = *p* > 0.05, *p < 0.05, **p < 0.01, ****p < 0.0001. Data combined from two experiments.

By the age of 10 weeks, BALB-neuT female mice spontaneously develop lesions in the mammary glands that give rise to metastases in the lung and bone marrow.^39^ Multiple tolerogenic mechanisms have previously been found to dampen humoral and cellular immunity to Her2 induced by the rEC-TM DNA vaccine.^40^ T regulatory cells were involved and found to increase in number with tumor progression.^40^ We used this stringent BALB-neuT model, in which both tolerance to Her2 and spontaneous cancer development challenge the immune response, to test our rED44Her2-containing vaccines. BALB-neuT mice were vaccinated once every 3 weeks, starting at 10 weeks of age, and their survival followed. Both the rED44Her2 and rEC-TM DNA vaccine extended survival by 3 weeks only compared to the control vaccine. In contrast, the rED44Her2-FrC conjugate performed significantly better than the other two vaccines (*p* < 0.0001 vs. either rED44Her2 or the rEC-TM DNA vaccine, Fig. 2A) with 50% of the mice surviving long term (until experiments were terminated after 20 weeks).

Since our vaccination protocol was focused on antibody induction antibody levels were then compared in detail. In contrast to the experiments in wild type (wt) mice where both rED44Her2 vaccines induced similar levels of antibody (Fig. 1B), in the tolerant model the rED44Her2-FrC conjugate vaccine primed significantly higher levels of antibody than rED44Her2 (Fig. 2B; *p* = 0.0152). The difference was more pronounced after the first booster injection, with average antibody levels in the rED44Her2-FrC group more than 3 times higher than in the rED44Her2 group (Fig. 2B; *p* = 0.0087). Although antibody levels equalized after the second boost the conjugate vaccine clearly demonstrated faster kinetics. At time points later than week 8 antibody levels could not be compared as mice in all groups except the rED44Her2-FrC group had died. The rEC-TM DNA vaccine induced significantly lower levels of anti-rED44Her2 antibody then either of the rED44Her2 containing vaccines at all three timepoints (Fig. 2B), similar to the data in wt mice (Fig. 1B).

### The rED44Her2-FrC conjugate vaccine induces CD4^+^ T cell help of a high magnitude and wide range, and hence induces high-quality antibody immunity

We next probed the ability of the two rED44Her2 vaccines to induce CD4^+^ Th cells in BALB-neuT mice. Mice were vaccinated with rED44Her2, rED44Her2-FrC or the control vaccine, all in alum, and specific Th cells were enumerated after *in vitro* restimulation with rED44Her2, FrC or a control protein. ELISPOT was used to detect Th cells secreting cytokines IL-2, IL-4 or IFNγ.

**Figure 3.**
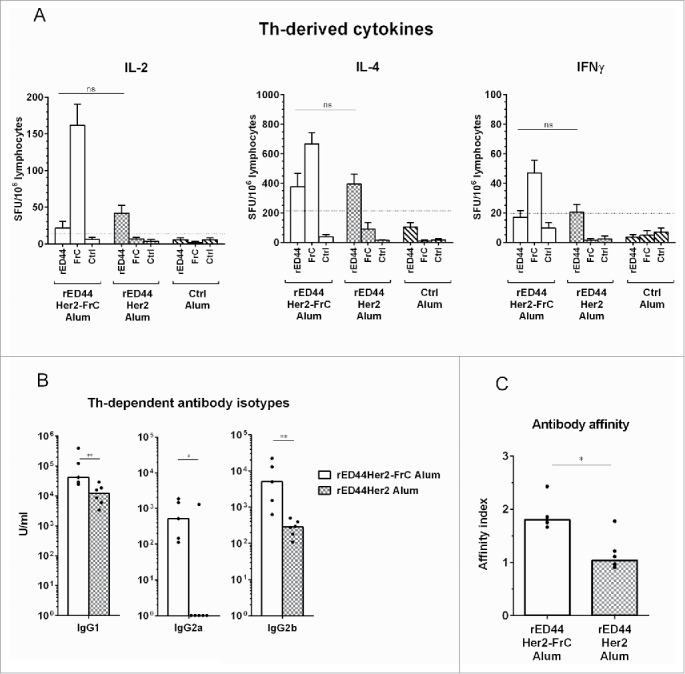
The rED44Her2-FrC conjugate vaccine induced potent CD4^+^ Th cell responses in BALB-neuT tolerant mice, which correlated with induction of a broad range of IgG antibody isotypes, and antibody of high affinity. (A) Tolerant BALB-neuT mice were vaccinated with either the rED44Her2-FrC conjugate vaccine, the rED44Her2 vaccine or a control vaccine (3–5 mice per group from three independent experiments). Spleens were taken at day 14 and the number of CD4^+^ Th cells secreting cytokines IL-2, IL-4 or IFNγ were measured by ELISpot after restimulation with rED44Her2 protein, FrC protein or an irrelevant control protein (Ovalbumin). The mean spot forming units (SFU) per 10^6^ lymphocytes (with SEM) from each group of mice is plotted, with non-specific background values (restimulation with media alone) subtracted. The cut-off line represents 2 times responses from mice vaccinated with the control vaccine, or responses to the control protein, whichever was highest. Mann–Whitney statistics are shown, between rED44Her2 stimulated groups. Ns = *p* > 0.05. (B) Antibody isotypes (IgG1, IgG2a and IgG2b) were measured by ELISA in BALB-neuT tolerant mice at week 5, following both priming and boosting with the rED44Her2-FrC or rED44Her2 vaccines, in alum. (C) Antibody affinity was measured using a chaotropic ELISA. Affinity index indicates the concentration of chaotropic agent required to reduce antibody binding by 50%, with higher affinity antibody requiring a higher concentration to disrupt binding. Serum was taken at week 5, as in (B). In (B) and (C) medians are plotted with data pooled from three independent experiments with similar results. Mann–Whitney statistics are shown, with *p < 0.05, **p < 0.01.

The rED44Her2 vaccine induced low numbers of IL-2 and IL-4 secreting Th cells, and the number of IFNγ secreting cells only just reached the threshold (Fig. 3A). These Th cells were rED44Her2-specific and not FrC-specific, as expected. Conjugation to FrC did not improve the magnitude or range of rED44Her2-specific Th cells (*p* > 0.05), but did induce large numbers of FrC-specific Th cells secreting the three cytokines. This implies the increase in antibody levels seen with the conjugate vaccine (Fig. 2B) was due to the improved Th response induced by the rED44Her2-FrC vaccine. Of note, in wt BALB/c mice the rED44Her2 vaccine was able to induce high numbers of rED44Her2-specific Th cells secreting the three cytokines (Fig. S2A)which suggests the failure in BALB-neuT mice was due to Her2 tolerance.

T-cell help guides antibody isotype switch, with a clear link between Th-derived IFNγ and IgG2a, and between IL-4 and IgG1.^41^ We found the poor ability of the rED44Her2 vaccine to induce IFNγ in tolerant mice resulted in the almost complete lack of vaccine-specific IgG2a induced (1/6 mice positive, Fig. 3B). The rED44Her2-FrC conjugate vaccine did induce IgG2a, in keeping with Th IFNγ induction. Concurrent with the reduced number of IL-4 secreting Th cells, less IgG1 was found with the rED44Her2 vaccine in comparison with the rED44Her2-FrC vaccine (Fig. 3B; *p* = 0.0087). With the Th-dependent isotype IgG2b, higher levels were again induced by the rED44Her2-FrC vaccine. As before, in the wt BALB/c mice the rED44Her2 vaccine could induce all three Th-dependent antibody isotypes, similarly to the conjugate vaccine (Fig. S2B).

Given the pivotal role of T-cell help in antibody affinity maturation, we next compared the affinity of the induced antibody using a chaotropic ELISA.^42^ Higher affinity antibody is less affected by the chaotropic agent, therefore, more remains bound to the plate. We found the conjugated rED44Her2-FrC vaccine induced significantly higher affinity antibody than the unconjugated vaccine (Fig. 3C; *p* = 0.0173).

Hence through the FrC portion, the rED44Her2-FrC conjugate vaccine was able to induce high levels of Th cells which secreted a wider range of cytokines than the unconjugated rED44Her2 vaccine. This resulted in not only higher levels of antibody (Fig. 2B) but in also in higher antibody affinity and multiple IgG isotypes.

### Foreign T-cell help is a critical component to overcome tolerance and induce potent antibody

We next investigated whether T-cell help could be substituted by other stimuli such as adjuvants which can activate B cells directly. Monophosphoryl lipid A (MPL) is a known LPS-derived ligand for toll-like receptor 4 (TLR4), clinically approved for use in combination with alum in the prophylactic vaccine against human papilloma virus (HPV) (Cervarix).^43^ MPL was also included in the clinical trial using a Her2 protein vaccine consisting of the EC domain plus a portion of the IC domain.^18^ We evaluated the rED44Her2 vaccine in combination with MPL plus alum and compared responses to the unconjugated or conjugated vaccine with alum alone, to see whether adding the MPL adjuvant could compensate for the lack of T-cell help. In a separate group MPL was added to the rED44Her2-FrC vaccine in alum, to test whether further improvement of this vaccine could be achieved.

**Figure 4.**
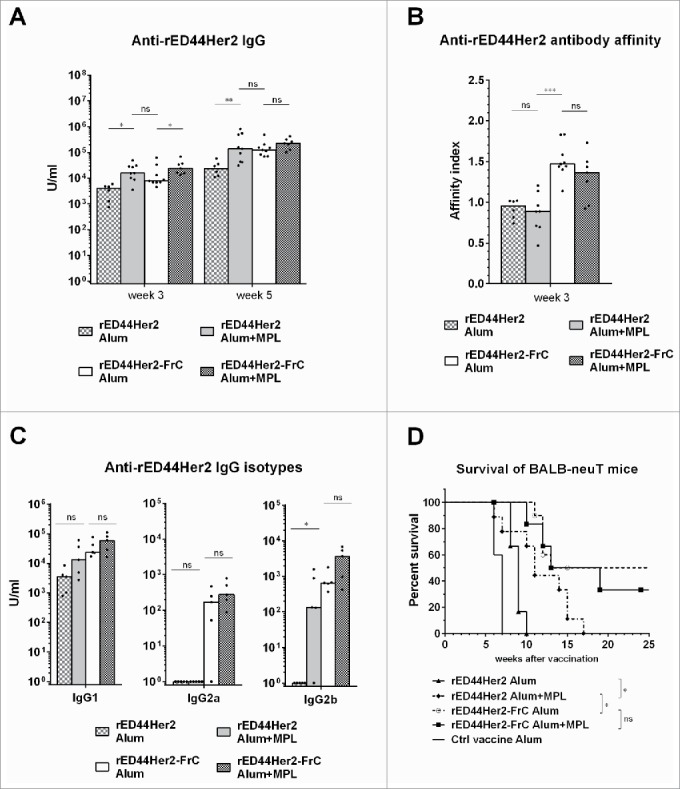
TLR4 agonist MPL cannot compensate for lack of T-cell help in terms of antibody affinity, IgG isotype induction or protection from spontaneous tumor development. BALB-neuT mice (n = 3–5 per group) were vaccinated with either rED44Her2 or rED44Her2-FrC, in alum or in combination with alum plus MPL. Serum was taken after priming, at week 3, or after boosting, at week 5. (A) Anti-rED44Her2 antibody levels measured by ELISA. (B) IgG antibody affinity from week 3 samples was measured by a chaotropic ELISA, and the affinity index calculated. (C) Levels of IgG isotypes were measured from serum samples taken at week 5. Medians are plotted, with Mann–Whitney statistics shown. Ns = *p* > 0.05, *p < 0.05, **p < 0.01, ***p < 0.001. (D) Survival of BALB-neuT mice from the spontaneous development of mammary tumors was followed after the above vaccination protocol. Mice were culled when total mean diameter of the tumors reached 15 mm. Log-rank (Mantel–Cox) statistics shown. Ns = *p* > 0.05, *p < 0.05. Due to the limited number of BALB-neuT mice available, results from two experiments showing the same trends have been combined in (A), (B), (C) and (D).

Fig. 4A shows that after priming (at week 3) inclusion of MPL did enhance antibody levels above that induced with rED44Her2 in alum alone (*p* = 0.028). The difference was even more apparent at week 5 (after boosting, *p* = 0.0048). Antibody levels induced by rED44Her2 in alum plus MPL were comparable to those in the rED44Her2-FrC group (*p* > 0.05), indicating that in terms of antibody levels MPL could replace T cell help. However, when we measured antibody affinity it became clear that addition of MPL did not yield any improvement in affinity above that in the rED44Her2 plus alum group (*p* > 0.05, Fig. 4B), and the affinity was significantly lower than in the rED44Her2-FrC conjugate vaccine group (*p* = 0.0002). Hence the enhancement of antibody levels in rED44Her2 plus MPL group was because of an increase in low-affinity antibody. MPL also failed to enhance the levels of IgG1 or IgG2a when added to the rED44Her2 vaccine (Fig. 4C), but did increase IgG2b levels (*p* = 0.048). In terms of impact on protection, MPL provided modest improvement of survival over that by the rED44Her2 vaccine in alum alone (Fig. 4D; *p* = 0.0413), but was less effective than the conjugate vaccine in alum alone (*p* = 0.0358). Finally addition of MPL failed to make any difference to the overall performance of rED44Her2-FrC vaccine (Figs. 4A–D).

### Performance of an ED44Her2-FrC clinical candidate vaccine

**Figure 5.**
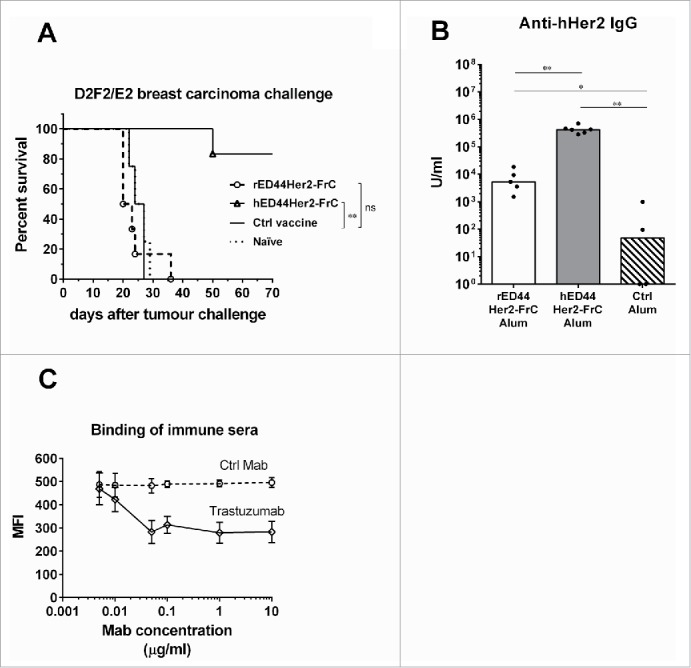
Evaluation of the rat and human ED44Her2-FrC conjugate vaccines in the D2F2/E2 model which expresses hHer2. (A) Wild type BALB/c mice (n = 4–6 per group) were vaccinated twice with the vaccines indicated (at week 0 and week 3) and at week 8 were challenged with 5 × 10^5^ D2F2/E2 mammary carcinoma cells. Survival was followed from the day of tumor challenge, with mice culled when total tumor diameter reached 15 mm. Log-rank (Mantel–Cox) statistics are shown, ns = *p* > 0.05, **p < 0.01. (B) Levels of anti-hHer2 antibody were quantified by analysis of binding to native hHer2 expressed on the surface of D2F2/E2 cells by FACS. Quantification was relative to internal standards. Bars are median values, with Mann–Whitney statistics shown, *p < 0.05, **p < 0.01. C. Competition assay in which binding of sera from hED44Her2-FrC-vaccinated mice to hHer2 on D2F2/E2 cells was detected by FACS in the presence of varying concentrations of trastuzumab or control Mab (rituximab). Mean values with standard deviations from groups of four individual mice are shown. Data are representative of three (A) and (B) or two (C) independent experiments.

The next step was to identify a candidate vaccine for clinical testing. Results generated in the BALB-neuT model suggest the rED44Her2-FrC conjugate is clearly more potent at protecting against cancer than the unconjugated rED44Her2 vaccine (Fig. 2A). Vaccines containing rat Her2 fragments have been proposed as xenogeneic vaccine candidates for human use.^44^ Therefore, we tested the rat ED44Her2-FrC in the transplantable D2F2/E2 model of breast cancer, which expresses human Her2 (hHer2). Wt BALB/c mice were vaccinated with rED44Her2-FrC using the same protocol as for the TUBO model (at week 0 and week 3) and then at week 8 they were challenged with the D2F2/E2 tumor. No protection was induced by the rat version of the vaccine in comparison with the group which received the control vaccine (*p* > 0.05, Fig. 5A). This was in keeping with low levels of antibody able to bind native hHer2 on the surface of D2F2/E2 cells, as measured by FACS (Fig. 5B). We then used the human version of the ED44Her2-FrC conjugate vaccine in the D2F2/E2 model and this induced significant protection, with approximately 80% of mice remaining tumor free until the experiment was terminated 70 d after tumor challenge (Fig. 5A; *p* = 0.0018). Protection was in keeping with higher levels of anti-hHer2 antibody, measured by FACS using D2F2/E2 cells (Fig. 5B; *p* = 0.0043).

Since the hED44 contained the region of Her2 that is recognized by trastuzumab, we were curious whether our vaccine induced reactivities similar to trastuzumab. We conducted a Her2 binding assay in the presence of trastuzumab, pertuzumab or control Mab. Trastuzumab-like reactivity was induced by the hED44Her2-FrC vaccine because trastuzumab but not the control Mab was able to block hHer2 binding of the immune sera (Fig. 5C). Since the immune sera still retained binding to Her2 even at saturating concentrations of trastuzumab (1 μg/mL), reactivities directed to other epitopes were clearly present. Pertuzumab which binds to Her2 outside of the hED44 region^45^ did not prevent binding of the immune sera as expected (Fig. S3). The combination of the two Mabs showed similar effects to trastuzumab alone (Fig. S3).

## Discussion

Here we propose a clinically relevant strategy for inducing protective antibody against Her2, using a conjugate protein vaccine in which a novel Her2 fragment is conjugated to the FrC carrier. To facilitate transition into the clinic, we used the magnICON system for vaccine expression and preparation.^30^ This innovative approach assures fast and cost-effective antigen production^46^ which was made possible via the use of plant-viral vectors to drive gene expression in non-transgenic plants.^30^ A plant-based approach has recently been used for production of the rat Her2 EC domain and, in a preliminary study, was shown to be, effective at inducing protective antibody in wt mice.^47^ In our study, we reduced the size of the EC domain to a smaller fragment (ED44), a strategy that often leads to higher levels of expression.

The magnICON approach has been validated for delivery of clinical grade cancer vaccines and antibodies. In the Phase I, clinical trial for non-Hodgkins lymphoma magnICON manufacturing produced patient specific vaccines targeting idiotypic antigen.^48^ In addition, during the 2014 West African Ebola outbreak, three humanised Mabs against the Ebola virus (ZMapp) were manufactured using magnICON and have been successfully used to treat infected patients.^49^ Although no toxicity has been demonstrated with these plant-expressed biologics *per se* the ability to raise a strong polyclonal antibody response against Her2 raises the question of potential cardiotoxicity which has been documented with trastuzumab treatment in patients with Her2 positive breast cancer.^50^ This may be linked to transient Her2 expression upon combination with anthracycline therapy.^51^ Interestingly no increase of cardiac adverse events was observed in patients when two Mabs pertuzumab and trastuzumab were combined,^52^ indicating that targeting more than one epitope may in fact be advantageous. However, since vaccine-induced antibody is more difficult to withdraw, this aspect will need cautious assessment.

Multiple layers of tolerance to Her2 exist in patients, which vaccination will need to overcome to induce protective immunity through antibody. In the BALB-neuT model, tolerance similarly affects the CD4^+^ T-cell and B-cell compartments. Central tolerance of CD4^+^ T-cells is incomplete and peripheral tolerance deepens as the tumor grows and the frequency of regulatory T-cells increases,^40^ conceptually reproducing events that govern failure of immune control in patients. In the Balb-NeuT model, the major protective mechanism is antibody as a number of vaccines including the “gold standard” DNA vaccine failed to protect in antibody deficient mice (Balb-NeuTμKO).^38,53,54^ Hence, the function of tumor-antigen-specific B cells is a critical factor for anti-Her2 vaccination. In the BALB-neuT model, the B-cell compartment will be subjected to tolerogenic mechanisms which operate to silence self-reactive B cells.^55^ These include deletion or functional silencing (anergy), which depends on whether antigen is cell-surface bound or present in a soluble form, respectively.^55^ Since in the BALB-neuT model Her2 is cell-surface expressed but also present in a soluble form, involvement of both deletion and anergy is expected.

**Figure 6.**
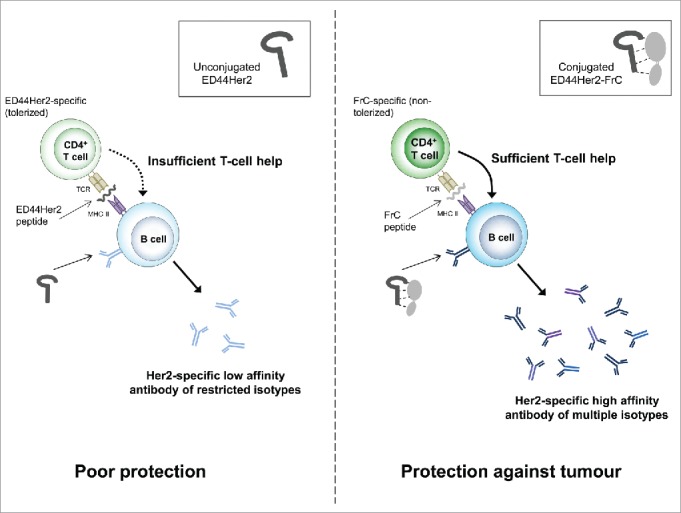
Proposed mechanism of action for the ED44Her2-FrC conjugate vaccine versus the unconjugated vaccine.

It is therefore remarkable that despite these tolerogenic mechanisms unconjugated vaccines were still able to induce antibody; however, the induced antibody was suboptimal and provided poor protection. We demonstrate that provision of sufficient T-cell help through conjugation to FrC was able to overcome tolerance and induce better quality antibody than the unconjugated vaccine: antibody of higher affinity and multiple IgG isotypes was induced (Fig. 6). The unconjugated vaccine induced low levels of Th cells which secreted IL-2 and IL-4, while cells that secreted IFNγ were almost undetectable. Conjugation to FrC was able to improve Th induction by engaging FrC-specific subsets able to secrete the three cytokines.

Our data suggest that the presence of FrC in the conjugate vaccine supports class switching to IgG2a. IgG2a antibody, or its human analog IgG1, is crucial for cytolysis of cancer cells due to superior binding of Fc receptors on innate immune cells which facilitate phagocytosis and antibody-dependent cell-mediated cytotoxicity. IgG isotypes are unlikely to be relevant for antibody-mediated direct inhibition of tumor cell growth (for example by down-modulation of Her2 signaling) but antibody affinity is expected to be critical. We found a significant increase in antibody affinity with the rED44Her2-FrC vaccine compared to the unconjugated vaccine. Our data suggest that the combination of both more desirable antibody isotype and higher affinity delivers the best outcome, with increased survival benefit found in the case of the conjugate vaccine.

Addition of adjuvant MPL to the unconjugated rED44Her2 vaccine contributed to an overall increase of anti-Her2 antibody, but was not linked to increased antibody affinity. This indicates that if insufficient T-cell help is available the extrafollicular pathway for antibody generation^56^ prevails over the germinal center pathway, and the quantitative increase in antibody following MPL addition was accounted for by low-affinity antibody. A clinical study using a dHer2 (EC plus IC portion) protein vaccine not conjugated to a carrier but combined with a complex adjuvant that included MPL, found that induced antibody was not able to sufficiently inhibit Her2 *in vitro*.^19^ Although affinity results were not reported, our data suggest this outcome could have resulted from induction of low-affinity anti-Her2 antibody.

It appears that inclusion of large protein carriers is superior to individual helper epitopes. For example, inclusion of the p30 universal helper epitope from FrC into a Her2-EC protein vaccine did not result in significant protection in the FVB/N^neu^ spontaneous breast cancer model.^57^ This is most likely because a large protein can engage a wider CD4^+^ T cell repertoire and so deliver larger quantities of T-cell help. The quantitative element of T-cell help is important and a known limiting factor for generating high-affinity antibody.^20^ In the clinic a single helper epitope, even if promiscuous for multiple MHC II alleles, is unlikely to be effective in an unselected patient population. For maximal antibody induction, we selected the full-length FrC sequence which we have shown to be effective with a range of antigens.^25^ The sequence consisting of a single-domain Dom of FrC was designed for vaccines aimed to induce CD8^+^ T-cell responses against attached peptide sequences^58^ and was not used here. Rat Her2 has more than 80% homology to the human molecule,^59^ and both rat and chimeric versions of the EC-TM DNA vaccine have been used to generate immunity against human Her2 positive tumors.^44^ Therefore, the rat version of the conjugate vaccine (rED44Her2-FrC) was tested for the ability to protect against the hHer2^+^ D2F2/E2 breast carcinoma. rED44Her2-FrC was not able to protect mice from D2F2/E2 tumor challenge, hence a human version of the ED44Her2-FrC vaccine was developed. This version was excellent at inducing hHer2-specific antibody and protected 80% of mice in the prophylactic model. Remarkably hED44Her2-FrC was able to induce trastuzumab-like reactivities but also targeted unknown epitopes of hHer2. As immune effectors, antibodies have proven powerful at achieving remarkable antitumor efficacy and appear less affected by tolerogenic mechanisms operating in the tumor microenvironment, particularly in solid tumors.^2^ This reassures that optimally-induced humoral immunity could be a very powerful antitumor therapy. The formulation of alum and MPL was previously demonstrated to induce functional CTLs.^32^ However, in our study no additional protection was observed when MPL, was added to the vaccine formulation, arguing that antibody is the necessary and sufficient for protection. Although co-induction of cytotoxic T cells is desirable, the ability of this arm of the immune response to control the tumor appears to encounter difficulties linked to the immunosuppressive microenvironment in the tumor itself.

Induction of powerful polyclonal antibody without the cytotoxic T-cell arm of the immune response could be sufficient for clinical success of the ED44Her2-FrC conjugate vaccine. However one can assume that multiple immune effectors will work synergistically and benefit the patient, although whether this is the case remains to be seen.

## Materials and methods

### Production of plant-derived vaccines

The rat ED44Her2 (rED44Her2) sequence (GenBank accession no. NP_058699, residues 314–657) was generated from rat cDNA by PCR and the human ED44Her2 (hED44Her2) sequence (GenBank accession number AAA75493, amino acid residues 310–653) was generated using gene synthesis with the codon usage of *Nicotiana tabacum*. The FrC sequence encoding the non-toxic C-terminal portion of Tetanus toxin, (Swiss-Prot accession no. P04958, amino acid residues 865–1315) was generated by gene synthesis.

rED44Her2, hED44Her2 and FrC protein was produced using magnICON methodology.^30^ For further details see supplementary material. Briefly, the DNA sequences were cloned into Tobacco mosaic virus (TMV) based viral expression vectors, then Agrobacterium containing the vectors were prepared as described^31^ and used to infiltrate *Nicotiana benthamiana* plants. 7–12 d later plant leaves were harvested, and the proteins purified by affinity chromatography. rED44Her2 and hED44Her2 were conjugated to FrC using glutaraldehyde, see supplementary material.

### Mice and vaccination protocols

BALB/c and BALB-neuT female mice, aged 10 weeks at the beginning of procedures, were kept in accordance with the UK Home Office Guidelines. BALB-neuT mice express the rHer2 oncogene under the mouse mammary tumor virus promoter on a BALB/c background,^36^ and spontaneously develop mammary tumors palpable by 12 weeks of age.

Mice were injected with 50 µg of rat or human ED44Her2, or the ED44Her2-FrC conjugates containing an equivalent amount of ED44Her2. At both priming and boosting the vaccines were combined with an equal volume of alum adjuvant (Sigma) and injected subcutaneously into two sites in the flank. In some experiments 10 µg per mouse of monophosphoryl lipid A (MPL, Invivogen) was added to alum as an additional adjuvant. An equivalent amount of plant-expressed irrelevant protein (idiotypic Ig from the 5T33 mouse myeloma fused to FrC,^21^ made by Icon Genetics) was used as a control vaccine. 50 µg of a DNA vaccine encoding the EC and TM portions of Her2 (Her2-EC-TM DNA vaccine^38^) was injected intramuscularly into both quadriceps. BALB/c mice were boosted 3 weeks after priming with the same amount of protein or DNA as at priming. BALB-neuT mice were vaccinated 6 times with a 3-week interval between vaccinations. The same amount of protein used at priming was used for the first boost, then half the amount was used for subsequent boosts.

### Mouse mammary carcinoma cell lines

TUBO cells were cloned from a lobular mammary carcinoma which developed in a BALB-neuT mouse, and therefore express rHer2.^60^ They were cultured in complete DMEM with high glucose (PAA Laboratories, with added 20% fetal calf serum (FCS), 50 U/mL penicillin and 50 µg/mL streptomycin).

The mammary tumor line D2F2/E2, which were derived from a BALB/c mouse and stably transfected with the human Her2 gene,^37^ were cultured in complete DMEM with high glucose, supplemented with 800 µg/mL of selective antibiotic G418 (Gibco). Single-cell suspensions were prepared by passing the cells through a syringe after trypsinisation. Both the TUBO and D2F2/E2 cell lines were a kind gift from Professor Lollini, University of Bologna, Italy.

### Measurement of Her2ED44-specific total IgG antibody, IgG isotypes and antibody affinity by ELISA

Nunc Maxisorp ELISA plates were coated overnight with 3 µg/mL rED44Her2 or hED44Her2. Following blocking with 1% bovine serum albumin (BSA) in phosphate buffered saline (PBS), serial dilutions of serum were incubated on the plate for 1.5 h at 37°C. Horseradish peroxidase (HRP) labeled anti-mouse IgG (The Binding Site), anti-IgG1 (Oxford Biotechnologies), anti-IgG2a (Serotec) or anti-IgG2b (Harlan Sera-Lab Ltd.) were added for a further 1.5 h at 37°C, followed by incubation with o-phenylenediamine dihydrochloride substrate (Sigma). Internal standards were generated as previously described^24^ and a standard curve used to calculate serum antibody levels.

Chaotropic ELISAs to measure antibody affinity followed the above protocol, with addition of chaotropic agent ammonium thiocyanate (serially diluted from 6M) following serum incubation.^42^ Binding of higher affinity antibodies to the coating antigen is less disrupted by the chaotropic agent, therefore more remain bound to the plate and are detected by the secondary antibody. The affinity index was calculated as the concentration of ammonium thiocyanate required to achieve a 50% reduction in bound antibody, compared to wells with no ammonium thiocyanate (100%).

### Antibody binding to native Her2

A FACS assay to measure the extent induced antibodies could bind to native Her2 expressed on the cell surface was modified from Rovero et al.^60^ Briefly, 2 × 10^5^ TUBO cells expressing rHer2, or D2F2/E2 cells expressing hHer2, were blocked with non-specific mouse serum then incubated with serial dilutions of serum from vaccinated mice. Binding of vaccine-induced antibody to native Her2 was detected using eFluor660-labeled polyclonal anti-mouse IgG (eBioscience). A standard curve was generated using pooled serum samples, and the relative binding of induced serum antibodies was estimated from this. An unlabeled monoclonal anti-Her2 antibody (Ab-4 for rHer2, Ab-5 for hHer2, both Calbiochem) was used as a positive control.

### Detection of trastuzumab-like reactivities in the immune sera

D2F2/E2 cells treated as for antibody binding were pre-incubated with trastuzumab, pertuzumab or negative control rituximab (all from Roche) Mabs at a range of increasing concentrations (0.001–10 µg/mL to reach saturating concentration) for 30 min on ice before individual or pooled sera from mice vaccinated with hED44-FrC was added and incubated for a further 30 mins. The mouse serum samples were obtained at week 7 from Balb/c mice vaccinated twice with hED44Her2-FrC as described above. Binding of the immune serum was detected using PE-labeled anti-mouse IgG Fc-specific antibody (Jackson ImmunoResearch, PA, USA) using FACSCalibur with Cell Quest Pro (BD Biosciences) and FlowJo (Tree Star Inc., Oregon, USA) software.

### Tumor protection experiments

In BALB/c mice protection experiments were performed using the TUBO (rHer2) or the D2F2/E2 (hHer2) cell lines. After two doses of vaccine (at week 0 and week 3) mice were challenged subcutaneously with 1 × 10^5^ tumor cells. The challenge was 4–6 weeks after the last vaccination. Once the tumors reached 15 mm mean diameter the mice were culled. In BALB-neuT protection experiments, culling took place when the sum total of tumor diameter reached 15 mm.

### ELISpot detection of vaccine induced Th cell responses

The ELISpot protocol for detecting Th responses after a protein vaccination was developed previously.^61^ Briefly, plates were coated overnight with anti-IFNγ, anti-IL-2 or anti-IL-4 antibody (BD Bioscience). 14 days after vaccination with rED44Her2, rED44Her2-FrC or the irrelevant control vaccine (plant expressed 5T33 Ig, not fused to FrC) spleens were processed. Following lymphoprep density centrifugation lymphocytes were plated at 2.5 × 10^5^ per well in RPMI 1640 media supplemented with 10% FCS, 1 mM sodium pyruvate, 2 mM glutamine, 1% non-essential amino acids, 50 U/mL penicillin, 50 µg/mL streptomycin and 50 µM β-mercaptoethanol. The cells were restimulated *in vitro* with media alone, 10 µg/mL rED44Her2, 10 µg/mL FrC or 10 µg/mL control protein (EndoGrade Ovalbumin, Hyglos GmbH). Following 40 h of restimulation, cytokine secretion was detected using cytokine-specific biotinylated antibodies, and then streptavidin-ALP (MAbtech AB). Spots were revealed using BCIP/NBT substrate (Invitrogen) and enumerated using an ELISpot microplate reader (AID GmbH). The non-specific media alone background was subtracted, and the response considered positive if the number of spots was more than twice that of the control mice or to the control protein, whichever was highest.

### Statistical analysis

Non-parametric Mann–Whitney statistics were used for comparison between groups, with *p* < 0.05 taken as significant. The Spearman's rank correlation coefficient, calculated using GraphPad Prism, was used to assess correlation between ELISA and FACS data. For curve comparison in survival experiments the Log-rank (Mantel–Cox) test was performed.

## Supplementary Material

KONI_A_1166323_supplementary_data.zip
