# Supplementary material for: A plant-expressed conjugate vaccine breaks CD4+ tolerance and induces potent immunity against metastatic Her2+ breast cancer
Source: Oncoimmunology. 2016 Apr 22;5(6):e1166323. doi: 10.1080/2162402X.2016.1166323 (PMC4938312; doi:10.1080/2162402X.2016.1166323)
Supplement: KONI_A_1166323_supplementary_data.zip [file koni-05-06-1166323-s001.zip › Supplementary material.docx]

**Supplementary Materials and Methods**

Production of the plant derived vaccines

During PCR cloning for the rat ED44Her2 (rED44Her2), or gene synthesis for human ED44Her2 (hED44Her2) and FrC sequences, flanking BsaI restriction enzyme sites were added and internal BsaI sites removed. These BsaI sites were then used to clone the DNA into Tobacco mosaic virus (TMV)-based viral expression vectors (magnICON system), as (1). The vectors contained a rice α-amylase signal peptide with the last two animo acids changed from the wild type sequence (MGKQMAALCGFLLVALLWLTPDVASG), to target the ED44Her2 or FrC proteins to the plant apoplast where it can accumulate. For purification purposes a (GGGGS)_3_ linker was added to the rHer2ED44, hED44Her2 and FrC sequences, then either a 6x His tag or the human kappa light chain constant region.

Agrobacterium containing the rED44Her2, hED44Her2 or FrC TMV vectors were used to infiltrate Nicotiana benthamiana plants, using the vacuum-infiltration protocols described in (2), in order to produce the recombinant proteins. After 7-12 days the plant leaves were harvested, and the rED44Her2, hED44Her2 and FrC proteins purified on affinity chromatography columns. Either a HisTrap FF column (GE Healthcare) for His tagged proteins or a KappaSelect column (GE Healthcare) for the kappa light chain tagged proteins was used. A SartobindQ SingleSep anion exchanger (Sartorius) and the EndoSafe-MCS system (Charles River Laboratories) were used to remove DNA, host cell proteins and endotoxin. Purified proteins were filter-sterilised using 0.2µm filters and kept at -80°C prior to use. The size and purity of the proteins were confirmed by SDS-PAGE.

Both rED44Her2 and hED44Her2 proteins were conjugated to FrC using glutaraldehyde. Equal amounts of ED44Her2 and FrC were incubated in 0.1% glutaraldehyde for 2 hours at room temperature. After the reaction was stopped by addition of 2M glycine, the conjugate ED44Her2-FrC was purified from excess glutaraldehyde and any non-conjugated proteins by gel filtration using a Superdex 200 column (GE Healthcare).

**References**

1. Engler C, Kandzia R, Marillonnet S. A one pot, one step, precision cloning method with high throughput capability. PLoS One. 2008;3:e3647.

2. Klimyuk V, Pogue G, Herz S, Butler J, Haydon H. Production of recombinant antigens and antibodies in Nicotiana benthamiana using 'magnifection' technology: GMP-compliant facilities for small- and large-scale manufacturing. Curr Top Microbiol Immunol. 2014;375:127-54.

**Supplementary Figure legends**

**Supplementary Figure 1**

Correlation between anti-rED44Her2 antibody measured by ELISA and anti-rHer2 antibody able to bind native protein expressed on the surface of TUBO cells, measured by FACS. BALB/c mice were vaccinated with rED44Her2-FrC in alum at week 0 and week 3, then serum collected at week 5. r= the Spearman’s rank correlation. Data combined from 2 independent experiments.

**Supplementary Figure 2**

The unconjugated rED44Her2 vaccine was able to induce T-cell help in BALB/c wildtype mice. (A) BALB/c mice were vaccinated with the rED44Her2 vaccine or a control vaccine, both in alum, then spleens taken on day 14. Splenocytes were restimulated *in vitro* with media alone, rED44Her2 or an irrelevant control protein (Ovalbumin) and CD4+ Th cells expressing IL-2, IL-4 or IFNγ were enumerated by ELISpot assay. The mean spot forming units (SFU) per 10^6^ lymphocytes from each group of mice is plotted, with SEM, after non-specific background values (restimulation with media alone) were subtracted. The cut-off line represents two times the responses from mice vaccinated with the control vaccine, or responses to the control protein, whichever was highest. Mann-Whitney statistics are shown, between rED44Her2 stimulated groups. *=p<0.05. (B) BALB/c mice were primed and boosted 3 weeks later with the rED44Her2-FrC or rED44Her2 vaccines, both in alum, then bled 5 weeks after the first injection. IgG antibody isotypes were measured by ELISA. Medians are plotted with Mann-Whitney statistics. ns=p>0.05. Data in (A) and (B) is from one of 3 representative experiments.
