# Supplementary figures and images for: A plant-expressed conjugate vaccine breaks CD4+ tolerance and induces potent immunity against metastatic Her2+ breast cancer
Source: Oncoimmunology. 2016 Apr 22;5(6):e1166323. doi: 10.1080/2162402X.2016.1166323 (PMC4938312; doi:10.1080/2162402X.2016.1166323)

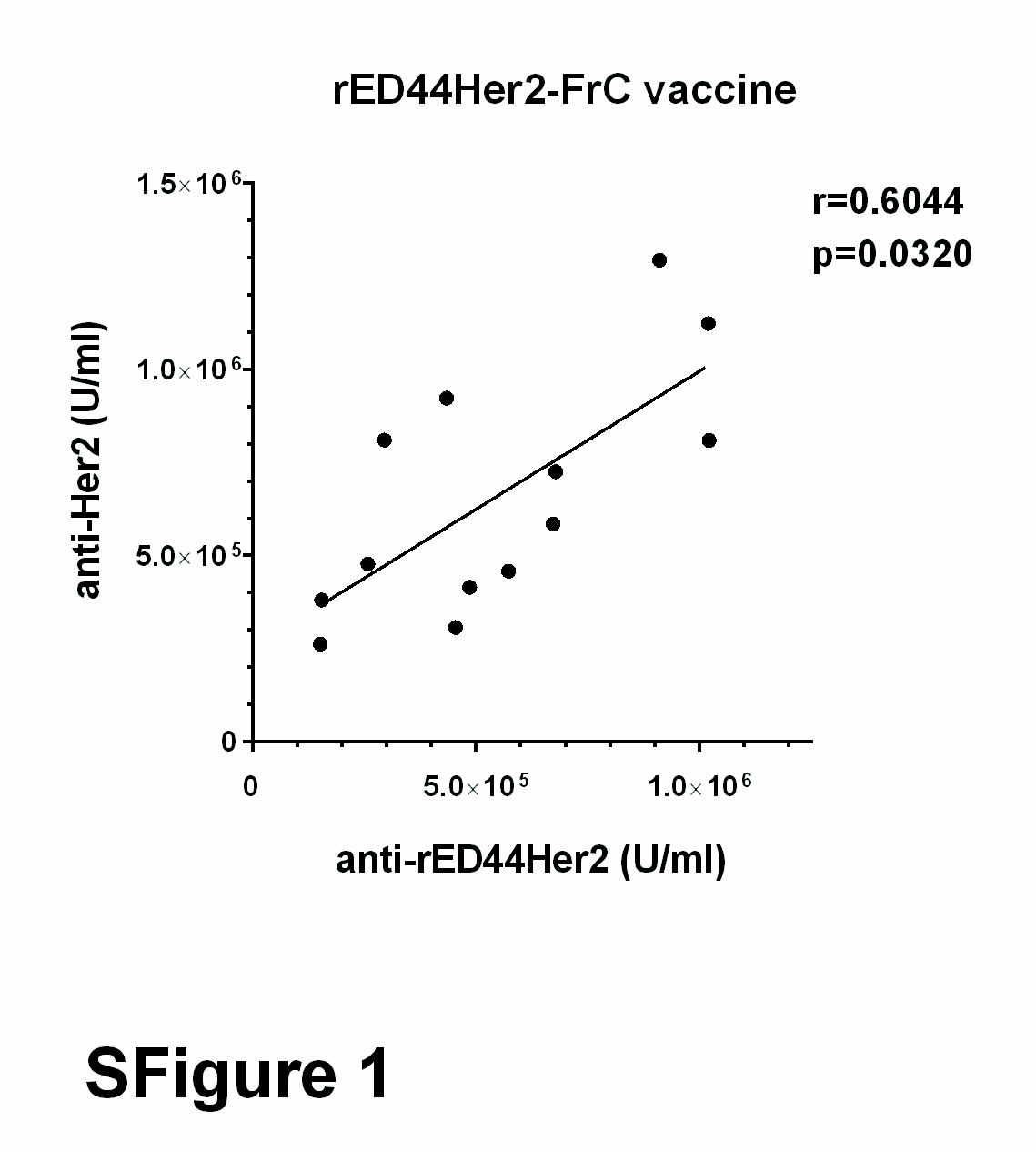

Supplement: KONI_A_1166323_supplementary_data.zip [file koni-05-06-1166323-s001.zip › KONI_A_1166323_s02.tif]

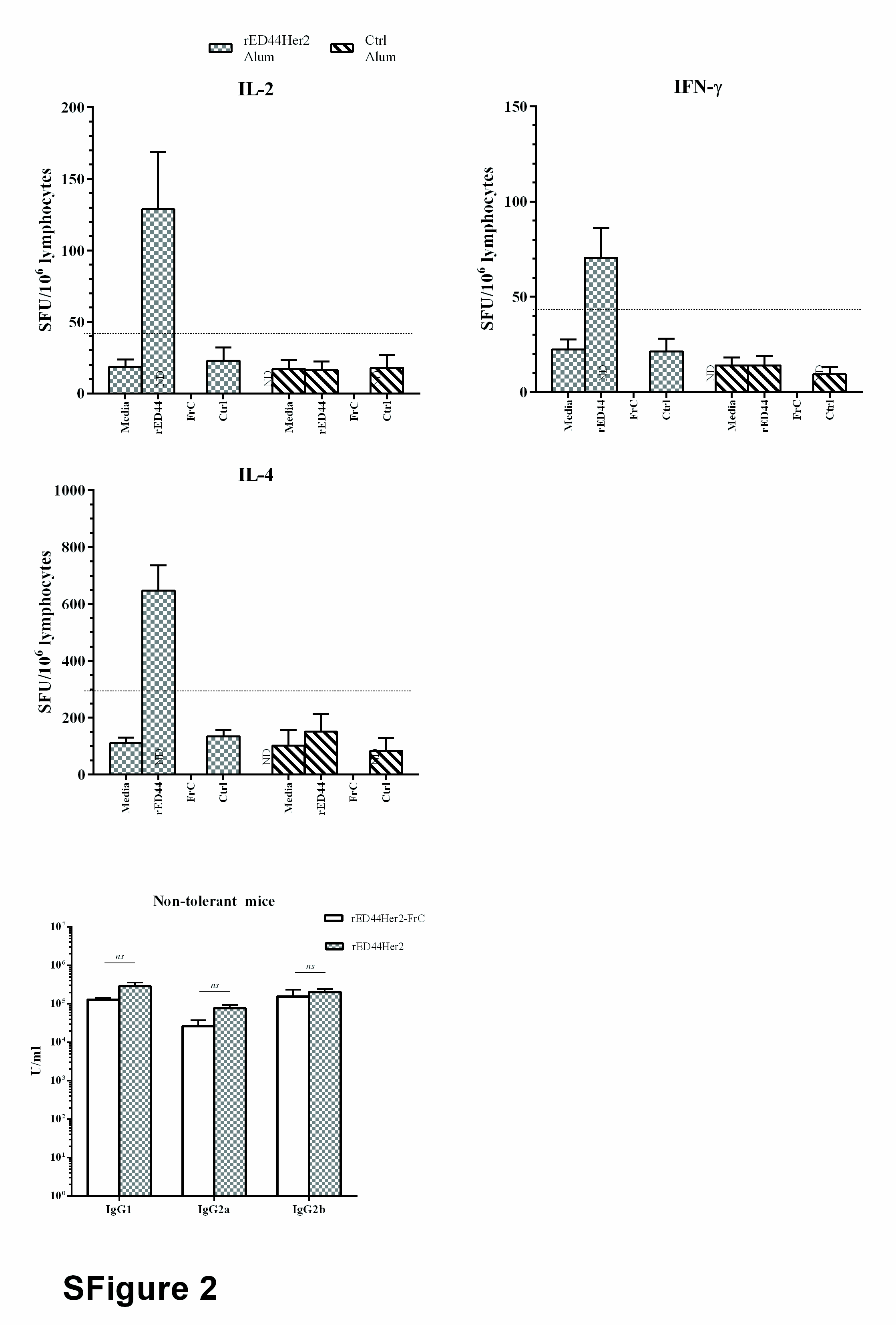

Supplement: KONI_A_1166323_supplementary_data.zip [file koni-05-06-1166323-s001.zip › KONI_A_1166323_s03.tif]

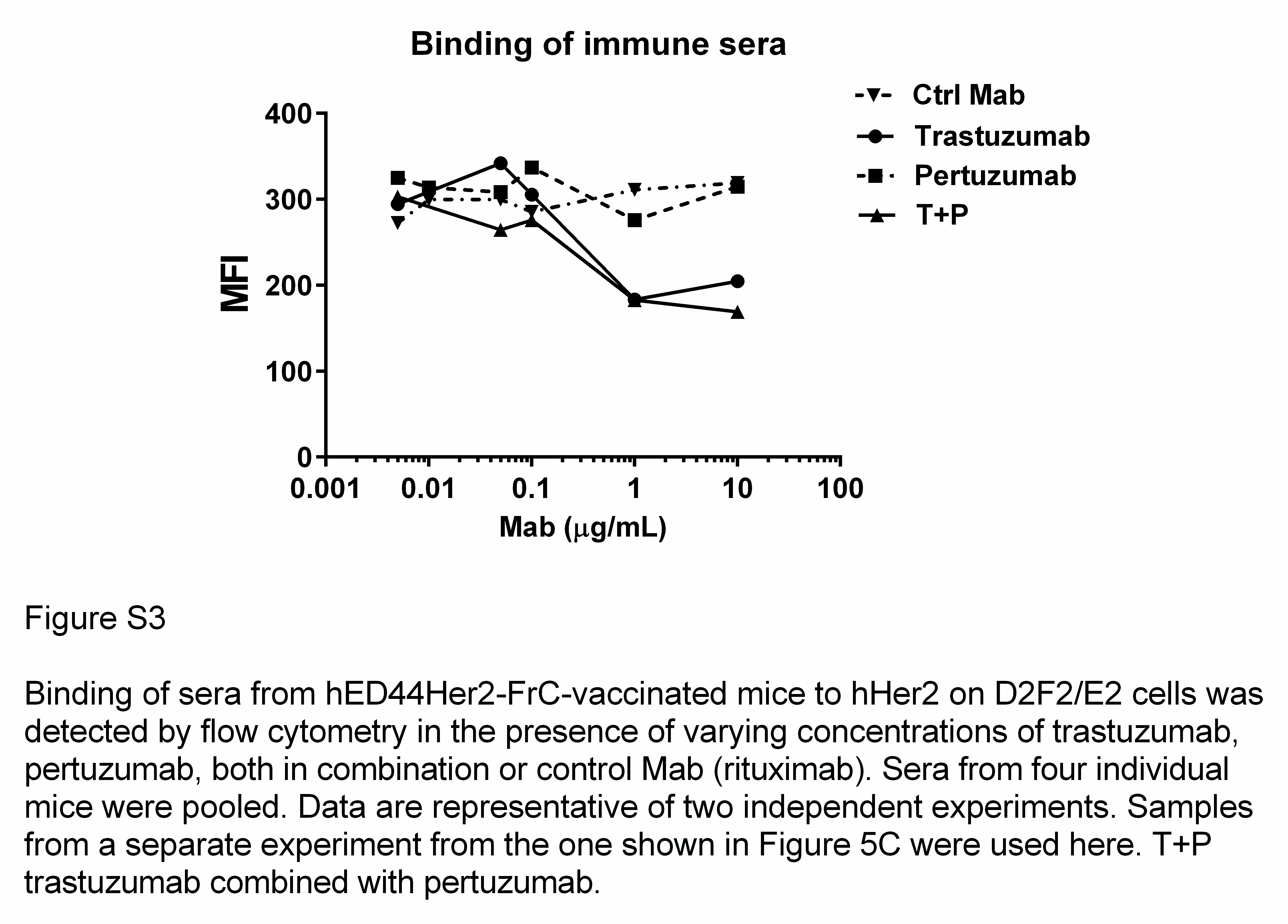

Supplement: KONI_A_1166323_supplementary_data.zip [file koni-05-06-1166323-s001.zip › KONI_A_1166323_s04.tif]
